# Five Cases of Extra-Genital Chancre

**Published:** 1897-04

**Authors:** Alfred E. Diehl

**Affiliations:** Buffalo, N. Y.; Lecturer on dermatology, Niagara Medical College; visiting dermatologist to the Erie County Hospital: dermatologist to the German Deaconess’s Home, etc. 362 Pearl Street


### FIVE CASES OF EXTRA-GENITAL CHANCRE.

By ALFRED E. DIEHL, M. D., Buffalo, N. Y.,

Lecturer on dermatology, Niagara Medical College; visiting dermatologist to the Erie County Hospital: dermatologist to the German Deaconess's Home, etc.

THE relatively great frequency of extra-genital chancres, occurring, as they do, so frequently on persons who are entirely innocent, leads me to report the appended cases and to say a few words concerning their diagnosis. First, as to their relative frequency: According to statistics collected by Julien, out of 1,977 chancres, 126, or over 6 per cent., were extra-genital; of these 126 cases, 73 were of the mouth, the frequency of the situation in the mouth being in the order of the lips first, then the tongue, gums and tonsils. According to statistics collected by Poray-Koschitz out of 852 extra-genital chancres 632 were cephalic. Of all these cephalic sores, I have reason to believe that a very large percentage occur on persons who are innocent; for example, many sores of the lips result from kissing and from contact with unclean drinking and eating utensils; those on the gums are generally due to unclean instruments employed by dentists.

Again, extra-genital chancres are much more frequently seen in some countries than in others; for example, Ricord put the proportion between extra-genital and genital sores as 1 to 3, the great majority of extra-genital sores being on women. In Vienna, while the proportion is not so great as in Paris, yet the ratio is very large. The same holds true in Russia. Bellonsow states that of all the cases he has seen, numbering nearly 3,000, only 25 per cent. were acquired during coitus. The reason that I quote these statistics is that many people come to the physician for treatment for some tertiary manifestation who have never had a primary genital sore and who deny any infection, thus showing that probably some extra-genital had been overlooked or mistaken for some other lesion ; the following secondary eruption had also passed unnoticed, as secondary signs in this country are often very mild and transient in form.

During the past year seven cases of extra-genital sores have come under my observation, of which number six persons were entirely innocent. Of the seven cases, two were on the finger, one on the eyelid, two of the tonsil, one on the abdomen and one on the instep. Two of the seven cases I will omit. The first case is as follows :

CASE I.—M. D., female, aged 17 years, came to me with general eruption, which proved to be a large papular syphilide. Examination of the genital region showed numerous condylomata lata, but no sign of an initial sore ; but on examining the mouth and throat, the left tonsil was seen to be much swollen and ulcerated. It appeared to be about the size of a small walnut, and was of a bluish-red color ; its surface was covered by a rather firmly adherent deposit of a grayish color. The tonsil itself was very much indurated, but caused very little pain. There was a general glandular enlargement, but the glands on the left side of the neck were considerably larger than those on the right.

CASE II.—C. H., male, aged 21, came to me complaining of a general eruption and sore throat. Examination revealed a macular eruption on the body. There was no sign of a genital sore. In the mouth the appearance was almost identical with that of Case I. The left tonsil was also affected in this case, but was not so large and was more deeply ulcerated, having a ragged edge and covered by a firmly adherent grayish-white deposit.

CASE III.—J. B., male, aged 7 years, whose father had died about two years ago, and the mother had married a second time and acquired a sore, which was followed by a macular eruption. I cautioned the mother to be very careful with her children and not to allow them to sleep in the same bed with the parents, nor eat with the same utensils. All went well for some time, when finally the mother brought in the boy, with a small sore on the right instep which refused to heal. The sore was about the size of a ten cent piece, superficially excoriated and very slightly indurated. There was some glandular enlargement in the popliteal space, but at the time there was no general glandular enlargement. In the course of a few weeks there appeared a macular eruption on the body. On inquiry as to how the boy became infected, it was found that the woman's husband, who had numerous condylomata lata in the gluteal fold, had been accustomed to place cloths there to keep the parts from contact with each other, and had left the cloths about the house promiscuously. The boy had picked up some of these cloths and had evidently got some of the virus on his hands, and, on going to bed, had most probably scratched his foot with the infecting finger and thus produced the lesion. I can account for the sore in no other way, as the boy had always slept alone and had never been allowed to go near the woman's husband.

CASE IV.—Male, aged 35 ; was bitten on the index finger. A small abrasion resulted—which refused to heal under ordinary treatment. In the course of a week or ten days the sore began to spread and to take on the peculiar induration. The nail became disfigured and finally came away. The tip of the finger was irregularly swollen, presented a raw, glazed ulceration about half an inch in diameter and was of a peculiar bluish tint. The induration was quite marked.

CASE V.—J. C., male, aged 30 ; had been going with an infected woman ; came to me with a sore on his abdomen. The sore was about the size of a penny, rather deeply ulcerated and presented no appreciable induration. There was the usual glandular involvement and a macular eruption just beginning to make its appearance. On inquiring as to how the sore happened to be in such a position, he said that while with the woman he had scratched a pimple on his abdomen, thus easily accounting for the presence of the sore.

Of all the seven cases mentioned, this last one was the only one in which the sore was not acquired innocently. The other six cases I have reason to believe were all innocent and purely accidental.

In the diagnosis of extra-genital chancres those of the tonsil present the greatest difficulty. In this situation it must be differentiated from several other affections, notably diphtheria, gumma, abscess, tuberculosis and epithelioma. In cases where it is possible to have an examination made by a skilled bacteriologist, the diagnosis of diphtheria from chancre is, of course, very easy and simple, but in some cases where bacteriological examination cannot be made, the differential diagnosis is often very difficult, especially in the prodromal period of the secondary eruption, in which the local appearances and general symptoms present much similarity. In both affections there are fever, general malaise, nausea or anorexia, pain on swallowing and glandular enlargement ; locally there are present the redness and enlargement of the tonsil and an adherent yellowish deposit. The differential points are first in the coloration. In case of chancre the color is a peculiar bluish red, which is not present in diphtheria ; next there is the marked induration, which is also not found in diphtheria. As regards the membrane in diphtheria, it is generally found stretching over the fauces and soft palate, while in chancres it does not spread, but remains fixed at the seat of invasion, although cases have been recorded in which the deposit has spread. *e. g.* I will give a case reported by Flatan, of Germany, which is as follows : The tonsil was enlarged, dark-bluish red in color ; on the surface was an ulceration, which was covered by a yellowish membrane, in the middle of which were brown-black deposits. During two weeks, in spite of absence of fever and only slight pain, the condition was called diphtheria. In the course of a few days there was a similar swelling on the other tonsil, presenting also a similar membrane. The diagnosis was made only on the appearance of the secondary eruption ; another point of diagnosis is the enlargement of the cubital glands, which, by this time, have become involved in case of syphilis.

The diagnosis from gumma is made by the history of a previous sore—if possible to get such a history—although a large proportion of cases will deny ever having had a sore ; by the appearance of the ulceration, which presents usually a small crateriform opening, with undermined edges, the absence of the marked induration and the presence of severe pain on swallowing, and by the situation. Gummata, as a rule, make their appearance on the anterior or posterior pillars, while chancre involves the tonsil itself.

The diagnosis from abscess is usually comparatively simple. Abscess begins acutely with severe pain, and the resulting enlargement is usually soft and fluctuating and soon shows signs of pointing. Sometimes, however, the diagnosis is more difficult, for cases will occur in which the peritonsillar tissue becomes inflamed, swollen and edematous ; in such cases the sore will appear to be rather deeply ulcerated and with a raised edematous surface surrounding it.

Primary tuberculosis of the tonsil is rare ; the ulceration is superficial and covered with flabby granulations ; the edges are characteristic, having the peculiar eaten-out, ragged appearance, and surrounding the ulcerated surface are seen, grouped or isolated, miliary tubercles ; there is also severe pain on swallowing, and further examination will usually show signs of pulmonary disease. Epithelioma of the tonsil is also rare, and when found presents a rather deep ulceration, with raised edges, and is much indurated. There is no general glandular enlargement ; the patient complains of lancinating pains and difficulty in swallowing. Microscopic examination in cases of epithelioma will prove the diagnosis.

In all cases of extra-genital chancre the points enumerated can only make one suspect the nature of the condition, and the positive diagnosis can only be made on the appearance of the secondary manifestations.

362 PEARL STREET.

